# New Frontiers of Early Diagnosis of Pancreatic Fistula after Pancreaticoduodenectomy

**DOI:** 10.3390/jcm11216423

**Published:** 2022-10-29

**Authors:** Alessandro Coppola, Roberto Cammarata, Vincenzo La Vaccara, Tommaso Farolfi, Damiano Caputo

**Affiliations:** 1 Department of Surgery, Sapienza Università di Roma, 00185 Rome, Italy; 2 General Surgery, Università Campus Bio-Medico di Roma, 00128 Rome, Italy; 3 General Surgery, Fondazione Policlinico Universitario Campus Bio-Medico, 00128 Rome, Italy

Although the mortality at 90 days has declined, pancreaticoduodenectomy (PD) is an extremely complex surgical procedure, with a non-negligible rate of major postoperative complications [1].

Postoperative pancreatic fistula (POPF) represents the most common and feared complication in pancreatic surgery because it can lead to other complications, some of which can be lethal.

The literature reports a wide range of POPF rates, up to 40% of cases, despite technical improvements and the development of minimally invasive approaches [2].

Despite multiple efforts to predict the risk, prevent the formation, and mitigate the severity of POPFs, such fistulas remain the greatest contributor to major morbidity and mortality after PD. This complication, as defined by the International Study Group for Pancreatic Fistulas (ISGPF), is divided into two main groups: clinically irrelevant fistulas (e.g., biochemical leaks) and clinically relevant pancreatic fistulas (CR-POPFs) requiring postoperative management adaptations (i.e., grades B and C). The resulting leakage of pancreatic effluent can lead to significant morbidity characterized by deep organ space infections, hemorrhage, end organ failure, and even death [3].

Intraoperative POPF risk stratification is the first step in prevention and mitigation strategies in the event of high risk pancreas. The most widely used and validated tool for risk stratification is the fistula risk score (FRS), which combines four risk elements (the pancreatic parenchymal texture, disease pathology, pancreatic duct size, and intraoperative blood loss) to highlight risk groups [4].

In the postoperative setting, many parameters have been analyzed over time to obtain a reliable marker capable of facilitating the early diagnosis of POPF.

Several studies have shown that the amylase values in drains on postoperative days one and three are a reliable predictor of POPF, and they currently represent the method of choice in the surveillance of these patients in the postoperative setting [5].

In the case of suspicion of POPF, abdominal imaging is mandatory. An abdominal CT scan performs better than abdominal ultrasound in the detection of small abdominal collections. Two studies mainly investigated the role of CT in the diagnosis of POPF. Most of the predictive data concerned the size (>8 cm) and structural complexity of the collections [6].

Kinaci et al. evaluated the efficacy of ultrasonography (US) in the early postoperative period after PD in diagnosing POPF, finding low sensitivity and high specificity for the early diagnosis of POPF after PD, suggesting a role as a diagnostic complement rather than a first-choice method [7].

Although peripancreatic collections are more frequently observed in patients with postoperative pancreatic fistulas, not all peripancreatic collections are amylase-rich. Radiological variables should be analyzed in predicting the occurrence of POPF; however, to our knowledge, there are currently no known radiological variables with high accuracy for amylase-positive peripancreatic collections.

Some authors have proposed developing a predictive method for CR-POPF based on the union between the drain amylase levels (DALs) on the first and third postoperative days (POD1–POD3) and CT scan on POD3.

Patients with POD1 DALs ≥ 666 U/L were at higher risk of CR-POPF (*p <* 0.0001). POD3 DA values ≥ 252 U/L predicted 88% of CR-POPF cases. In addition, when POD3 DAL ≥ 207, the routine use of an abdominal CT scan on the same day could be useful for detecting abdominal collections ≥ 5 cm that were related to CR-POPF [2].

In addition, multiple markers, especially inflammatory markers, have been analyzed to identify some that could predict the onset of POPF.

In fact, POPFs are associated with inflammation that includes both that induced by pancreatic fluid and secondary inflammation induced by resulting bacterial infections, so inflammatory markers are expected to reflect the momentum of POPFs.

C-reactive protein (CRP) has also been shown to predict anastomotic leak after colorectal surgery. Studies assessing the role of CRP as a predictive factor for clinically relevant complications after PD have, however, shown mixed results, with some suggesting that CRP is a powerful predictor of CR-POPF and others questioning its predictive value.

Kanda et al. analyzed 153 patients undergoing proximal and distal pancreatectomy complicated by POPF and showed that the elevation of the CRP level, ≥28.4 mg/L, from POD1 to POD3, was an independent diagnostic factor for clinically relevant POPFs. The authors concluded that a steep rise in the serum CRP level from POD1 to POD3 was a highly predictive factor for subsequent clinically relevant POPFs [8].

Moreover, serum procalcitonin (PCT) has recently emerged as a potential predictor of intra-abdominal infections. PCT is a marker of severe bacterial infections and has the potential to distinguish between infectious and non-infectious systemic inflammation. PCT can also predict anastomotic leakage after colorectal surgery; however, PCT has not been extensively used as an early marker of complications after pancreatic surgery. To date, only a few studies have discussed the potential of PCT for predicting complications after PD.

Bianchi et al. conducted a small prospective study on 32 patients and showed that PCT on postoperative day (POD) 2 was the best predictor of infectious complications after PD [9].

More recently, Lei et al. retrospectively analyzed 86 pancreatectomy patients and demonstrated that PCT is valuable for the early prediction of pancreatic fistulas after pancreatectomy [10].

Giardino et al. demonstrated that PCT > 0.4 mg/dL on POD1 could be an early predictor of CR-POPF after pancreatoduodenectomies [11].

Caputo et al. combined the POD1-DAL and preoperative inflammatory biomarkers (PIBs)—such as the neutrophil-to-lymphocyte ratio (NRL), derived neutrophil-to-lymphocyte ratio (dNRL), and platelet-to-lymphocyte ratio (PLR)—in 227 patients who underwent PD, in order to predict grade C POPF. A POD1-DAL ≥ 807 U/L significantly predicted grade C POPF (sensitivity: 72.7%; specificity: 64.4%; AUC: 0.676; *p* = 0.004), with a PPV of 17.8% and an NPV of 95.6%. When PIBs were combined with a POD1-DAL ≥ 807 U/L, the PPV of grade C POPF rose from 17.8% to 89% [12].

Additionally, the bacterial flora could represent a risk factor for POPF. An intraoperative study of the biliary bacterial flora of patients undergoing PD was also performed. The prevalence of polymicrobial biliary cultures with *Escherichia coli*, *Klebsiella pneumoniæ*, *Enterococcus fæcalis*, and *Enterococcus fæcium* was significantly associated with POPF; therefore, antibiotic therapy tailored to the results of intraoperatively collected biliary samples may improve the outcomes in PD [13].

Among the markers proposed recently, we mention presepsine, a soluble CD14 subtype that is released into the circulation after the activation of the proinflammatory signaling cascade in response to infectious agents. Hiraki et al. published an interesting prospective pilot study showing that the concentrations of presepsine in drains were significantly higher in patients who went on to develop CR-POPF, suggesting that it may serve as an early marker in this context [14].

The volumes of the surgical drains have also recently emerged as a possible marker of POPF.

An interesting retrospective analysis was carried out by Fukui et al., analyzing 404 patients undergoing PD. They analyzed the predictive value of the drain output volume (DOV) for postoperative complications. A low DOV on POD1 was an independent predictor of CR-POPF. In their series, the optimal cut-off value of the DOV was 227 mL. This value was calculated with a receiver operating characteristic analysis that revealed that the DOV on POD1 had a negative predictive value, with an area under the curve of 0.655, sensitivity of 65.0%, specificity of 65.3%, and 95% confidence interval (CI) of 0.587–0.724.

They concluded that a low DOV on POD1 should alert surgeons, in order to avoid early drainage removal in patients potentially at high risk of CR-POPF [15].

In conclusion, the pathophysiological complexity of POPF makes its early diagnosis complex. Multiple markers have been analyzed to date; however, many of the results obtained conflict with each other and do not allow the validation of a method as a choice. Further efforts are needed to consolidate the data obtained to date, and at the same time, equal attention must be paid to the identification of additional markers able to predict the diagnosis and severity of POPF.
